# A Retrospective Research of the Characteristic of Hypertriglyceridemic Pancreatitis in Beijing, China

**DOI:** 10.1155/2016/6263095

**Published:** 2016-01-05

**Authors:** Wei-Ping Tai, Xiang-Chun Lin, Hong Liu, Cang-Hai Wang, Jing Wu, Neng-Wei Zhang, Wei Chen

**Affiliations:** ^1^Department of Gastroenterology, Beijing Shijitan Hospital, Capital Medical University Beijing 100038, China; ^2^Department of General Surgery, Beijing Shijitan Hospital, Capital Medical University, Beijing 100038, China; ^3^Department of Intensive Care Unit, Beijing Shijitan Hospital, Capital Medical University, Beijing 100038, China

## Abstract

*Aim*. To investigate the characteristic of hypertriglyceridemic- (HTG-) induced pancreatitis (HTG pancreatitis).* Methods*. We reviewed 126 cases of HTG pancreatitis and 168 cases of biliary pancreatitis as control.* Results*. The HTG group mean age was younger than biliary group. The number of females was a little higher than males in both groups. There were 18 cases that were recurrent in HTG group and 11 in billiary group. The mean hospitalization times were 13.7 ± 2.6 and 11.2 ± 2.3 days in two groups. Six patients received apheresis in HTG group. The proportion of severe AP was 31.0% and 26.2%, mortality 1.6% and 1.2%, comorbidity of diabetes mellitus (DM) 20.6% and 6.5% in two groups. The number of complications of gastrointestinal (GI) bleeding, sepsis, and multiple organ dysfunction syndrome (MODS) in HTG group and biliary group was 1, 1, and 2 versus 4, 12, and 4.* Conclusions*. The proportion of recurrent and severe AP and comorbidity of DM of HTG group was higher than billiary group. The proportion of the complications of GI bleeding, sepsis, and MODS of HTG group was less than biliary group. Apheresis could effectively reduce serum TG levels soon. There was no significant difference of the mortality between two groups.

## 1. Introduction

Acute pancreatitis (AP) represents an inflammatory reaction of the pancreas [1]. Due to the possibility of local or systemic complications, the patients are admitted to departments mainly for internal treatment. Patients who develop persistent organ failure within the first few days are at increased risk of death possibility [2–4].

HTG is a well established AP etiology and recurrent acute pancreatitis [2]. The recognition of HTG as a cause of AP is often delayed or missed [2]. Patients with HTG pancreatitis often have recurrent attacks and may require repeated hospitalizations. Good control of serum triglyceride (TG) levels can prevent the recurrences. In China and in the whole world, there is a trend that the proportion of HTG pancreatitis in the all AP is increasing overall [1, 2].

In this research we investigated the character of HTG pancreatitis in our hospital. The group of biliary pancreatitis were used as control. Informed consent was obtained from these patients to report their data upon approval of the Ethics Committee of our institution.

## 2. Materials and Methods

### 2.1. Patients and Methods

Between February 2010 and January 2014, 126 patients of HTG pancreatitis AP and 168 cases of biliary pancreatitis were enrolled into the our hospital (Department of Gastroenterology, Department of General Surgery, and Department of Intensive Care Unit of Beijing Shijitan Hospital of Capital Medical University). The patients were identified using primary diagnosis through computer-based system. The data with medical charts were reviewed retrospectively and the relative clinical data were collected. The diagnosis of acute pancreatitis requires two of the these features: (1) abdominal pain consistent with acute pancreatitis (acute onset of a persistent, severe, epigastric pain often radiating to the back); (2) serum amylase activity at least three times greater than the upper limit of normal; and (3) characteristic findings of acute pancreatitis through contrast-enhanced computed tomography (CECT) or magnetic resonance imaging (MRI) or transabdominal ultrasonography [5–8]. Serum triglyceride level more than 11.3 mmol/L (1000 mg/dL) and exclusion of other etiologies were recognized as the HTG etiology [9].

The finding of stone in common bile duct through CT or Magnetic Resonance Cholangiopancreatography (MRCP) could help to define the biliary etiology of acute pancreatitis and also should exclude other etiologies of AP [2, 5].

The severity of AP was classified into mild, moderate, or severe type according to the new Atlanta classification [1]. Mild acute pancreatitis had no organ failure or local or systemic complications and usually resolves in the first week. Moderate type was defined by the presence of transient organ failure, local complications, or exacerbation of comorbid disease relieved in 24 h. Severe type was defined by persistent organ failure (>48 h). Local complications were peripancreatic fluid collections, pancreatic and peripancreatic necrosis, pseudocyst, and walled-off necrosis [1]. Once admitted into hospital all patients were treated medically according to generally accepted principles including inhibiting oral intake, restoring fluid and electrolytes intravenously, and administration of prophylactic or treatment antibiotics [1]. A proton pump inhibitor was given to inhibit gastric acid secretion and somatostatin to inhibit pancreatic excretion.

Serum triglyceride (TG) level was checked shortly after admission. If there was evidence of severe AP, pancreatic rest via deeper ligament of Treitz nasojejunal enteral feeding tube [10] (preferred) or total parenteral nutrition (TPN) would be entertained [11].

Apheresis has been shown to remove TG from the serum of patients with HTG pancreatitis quickly [12–16]. The premise of using apheresis is that it removes available TG in very low density lipoprotein (VLDL) and chylomicrons from serum and prevents generation of FFA which cause local or systemic effects [17, 18]. In most patients, TG levels decrease by ~65% and 85% after one or two sessions, respectively [2]. There were 6 patients enrolled into apheresis treatment in HTG pancreatitis group and TG levels decreased more than 60% soon in our research. The candidates for apheresis were the patients with severe AP who continue to have TG levels above 1000 mg/dL after the first 24–48 hours [2].

Surgical intervention (necrosectomy with drainage or continuous postoperative lavage) was performed when infected pancreatic necrosis was clinically suspected or confirmed by positive bacteriologic results of CT-guided fine-needle aspirations. Endoscopic retrograde cholangiopancreatography (ERCP) was performed in 6 cases in biliary pancreatitis group. The indications included the following: (1) The patients had the clinical manifestation of cholangitis or biliary obstruction, (2) the patients' conditions aggravated, and (3) CT or MRCP showed that the stone of sludge exists in the common bile duct [4]. A papillotomy was done if stones and sludge were present in the common bile duct and some followed with endoscopic nasobiliary drainage.

Comorbidity was defined as a preexisting disease in addition to the acute pancreatitis. Comorbidity was diagnosed if the condition was an active problem and/or needs routine treatment prior to the onset of acute pancreatitis. The assessment of organ function and diagnosis of MODS was performed on the basis of the APACHEII multiple organ dysfunction score [14].

### 2.2. Statistical Analysis

Statistical evaluation was performed with SPSS 18.0 software. The significant differences of clinical characteristics of the HTG pancreatitis and biliary pancreatitis patients were tested with the *χ* ^2^ test. The differences between the two groups were tested through independent samples *t*-test. *P* < 0.05 was considered statistically significant.

## 3. Results

### 3.1. General Clinical Characteristics

Patient general characteristics are summarized in Table 1.

### 3.2. Comorbidity

Summarized in Table 2.

## 4. Discussion

HTG is a metabolic disorder due to an elevated synthesis of TG-rich lipoproteins VLDL, a reduced catabolism of these particles, or a combination of both mechanisms. Normally, values of TG are below 2.2 mmol/L (200 mg/dL) [19, 20]. The role of HTG in causing AP is commonly accepted. According to the literature, HTG is the third cause for acute pancreatitis after gallstones and alcohol [21, 22]. HTG is reported to account for more than 10% of all AP episodes [23]. There is even some evidence that HTG pancreatitis is associated with a higher severity and a higher complication rate [24, 25].

Several mechanisms for the disease have been indicated including the hydrolysis of TGs forming free fatty acids inducing inflammation, chylomicrons inducing hyperviscosity leading to ischemia, and genetic predisposition [26, 27]. In addition, cytokines seem to play an important role in acute pancreatitis including the systemic responses [28]. It is generally accepted that TG levels of > 11.3 mmol/L (1000 mg/dL) trigger AP and induce serious complications. Therefore, rapid lowering of TG levels is a primary medical goal in preventing serious harm to the patient suffering from HTG.

In our research the patients in HTG pancreatitis groups were younger than biliary pancreatitis group (35.7 ± 4.2 years versus 58.6 ± 5.6 years, Table 1). The ratio of male and female was close in HTG pancreatitis groups and biliary pancreatitis group (male 48.4% versus 48.8%, female 51.6% versus 51.6%). There were 2 patients (2/126, 1.6%) in HTP pancreatitis group and 4 patients (4/168, 2.4%) underwent surgical drainage in biliary pancreatitis group. There was no clear difference from the former reported research in these two types of AP. Maybe the reasons lied in the fact that biliary group had more serious infection and older age than HTG group. The sample of our research was not so big and our research is a retrospective research, so well designed prospective research is needed.

The medium hospital stay of HTG pancreatitis groups was significantly longer than biliary pancreatitis group (12.5 ± 2.6 days versus 9.3 ± 1.9 days). The reason was that the ratio of severe AP in HTG pancreatitis was higher than the biliary pancreatitis group (31.0% versus 26.2%).

The recurrent proportion in HTG pancreatitis in our research was 17.5% (22/126) and higher than biliary group (14.9%, 25/168). After detailed disease history inquiry, all the patients of 22 cases of recurrent in HTG pancreatitis groups have inducement. Ten patients had stopped the lipid lowering drugs and all the others were high fat diet.

The common comorbidity in HTG pancreatitis in groups was diabetes mellitus (26/126), hypertension (8/126), and coronary heart disease (3/126) as shown in Table 2. The comorbidity of DM in HTG pancreatitis group was evidently higher than biliary pancreatitis group (20.6% versus 6.5%) as shown in Table 2. There is report that the patients with HTG have more possibility of DM [24]. In Table 3 it showed that the proportion of severe AP in HTG pancreatitis group was higher than biliary pancreatitis group (31.0% versus 26.2%). The proportion of mild and moderate AP was 45.2% and 23.8% versus 42.9% and 30.9% (Table 3). The most frequent complication of AP in HTG pancreatitis group and biliary pancreatitis group was acute lung injury/acute respiratory distress syndrome (ALI/ARDS) (23.8%), followed by renal insufficiency (14.3%) and cardiovascular insufficiency (13.5%) as shown in Table 4. It was similar with the other reports from China [29]. In a group of enrolled 2461 patients of acute pancreatitis study in China, it showed that the total number was increasing year by year. The etiology included biliary (1372, 55.75%), alcoholism (246, 10%), hypertriglyceridemia (255, 10.36%), and the others (588, 23.89%). HTG pancreatitis increased at a faster rate than alcoholic AP and is the third important reason [29]. The complication of GI bleeding, sepsis, and MODS was just 1, 1, and 2 in HTG pancreatitis group and the ratio was also smaller than biliary group in our research. There was report from China that the GI bleeding in AP is mainly due to peptic ulcer and acute gastric mucosal lesions. There was no direct correction to the etiologies [30]. Maybe the sample of biliary pancreatitis bigger than HTG pancreatitis was one of the reasons. The sepsis and MODS mainly resulted from serious infection in AP patients. Also there was no direct correction to the various etiology [31]. So the proportion of sepsis and MODS in biliary group, higher than HTG group, may be the result of more serious infection and older age.

The mortality in HTG pancreatitis and biliary pancreatitis was similar (1.6% versus 1.2%). It is consistent with other reports [2].

In conclusion, for the tread of the increasing proportion of HTG pancreatitis in total AP, we should pay more attention to the characteristic and treatment of HTG pancreatitis. The proportion of recurrent, the proportion of severe AP, and the comorbidity of DM were higher than biliary group. The proportion of the complications of GI bleeding, sepsis, and multiple organ dysfunction syndrome (MODS) in HTG group was less than biliary group. Apheresis could effectively reduce serum TG levels soon. There was no significance difference of the mortality between two groups. Also there is shortcoming of our research, the total sample is not big, and it is a retrospective research. The number of aphereses is not big (6 cases) and needs more research. We do not discuss the detail type of HTG.

## Figures and Tables

**Table 1:** General clinical characteristics of patients with AP.

	HTG pancreatitis (*n* = 126)	Biliary pancreatitis (*n* = 168)	*P* value
Mean age (year, range)	35.7 ± 4.2	58.6 ± 5.6	<0.05
Male	61 (48.4%)	82 (48.8%)	—
Female	65 (51.6%)	86 (51.2%)	—
Medium hospital stay in days (range)	12.5 ± 2.6	9.3 ± 1.9	<0.05
Surgical drainage	2 (1.6%)	4 (2.4%)	<0.05
Recurrence rate	22 (17.5%)	25 (14.9%)	>0.05
Hospital deaths	2 (1.6%)	2 (1.2%)	>0.05

**Table 2:** Comorbidity in acute pancreatitis.

	HTG pancreatitis (*n* = 126)	Biliary pancreatitis (*n* = 168)	*P* value
Hypertension	8 (6.3%)	29 (17.3%)	<0.05
Coronary heart disease	3 (2.4%)	22 (13.1%)	<0.05
Diabetes mellitus	26 (20.6%)	11 (6.5%)	<0.05
Renal insufficiency	—	15 (8.9%)	—
Cerebral vascular disease		12 (7.1%)	

**Table 3:** The classification of AP.

	HTG pancreatitis (*n* = 126)	Biliary pancreatitis (*n* = 168)
Mild	57 (45.2%)	72 (42.9%)
Moderate	30 (23.8%)	52 (30.9%)
Severe	39 (31.0%)	44 (26.2%)

The classification of AP was according to the revised Atlanta standard [1]. The patients enrolled before 2013 were reevaluated according to the new standard.

**Table 4:** Organ failures and complications in AP.

	HTG pancreatitis (*n* = 126)	Biliary pancreatitis (*n* = 168)	*P* value
ALI/ARDS	30 (23.8%)	42 (25.0%)	>0.05
Renal insufficiency	18 (14.3%)	26 (15.5%)	>0.05
Cardiovascular insufficiency	17 (13.5%)	25 (14.9%)	>0.05
GI bleeding	1 (0.8%)	4 (2.4%)	<0.05
Sepsis	1 (0.8%)	12 (7.1%)	<0.05
With MODS	2 (1.6%)	4 (2.4%)	<0.05
